# Supplementary material for: Optimal inventorying and monitoring of taxonomic, phylogenetic and functional diversity
Source: PLoS One. 2024 Jul 31;19(7):e0307156. doi: 10.1371/journal.pone.0307156 (PMC11290677; doi:10.1371/journal.pone.0307156)
Supplement: S1 Table — At two sites (with *) 320 samples were performed and these were used only for the optimization of inventorying. At 16 sites, 24 samples were performed, and these were used only for the optimization of monitoring. (PDF) [file pone.0307156.s001.pdf]

S1 Table. Eighteen sampled sites in Iberian oak forests. At two sites (with *) 320 samples were performed and these were used only for the optimization of inventorying. At 16 sites, 24 samples were performed, and these were used only for the optimization of monitoring.

Site	Latitude	Longitude	Elevation (m)	Reference
**Aiguestortes 1**	42.5496	0.8725	1760	Crespo et al. 2018
**Aiguestortes 2**	42.5491	0.8714	1740	Crespo et al. 2018
*** Arrábida**	38.5067	-8.9752	60	Cardoso et al. 2008a
**Cabañeros 1**	39.3566	-4.3591	760	Crespo et al. 2018
**Cabañeros 2**	39.3516	-4.3589	740	Crespo et al. 2018
**Cabañeros 3**	39.3618	-4.4173	770	Crespo et al. 2018
**Cabañeros 4**	39.3634	-4.4170	770	Crespo et al. 2018
*** Gerês**	41.7953	-8.1363	660	Cardoso et al. 2008b
**Monfragüe 1**	39.8330	-6.0641	320	Crespo et al. 2018
**Monfragüe 2**	39.8280	-6.0325	320	Crespo et al. 2018
**Ordesa 1**	42.6068	0.1313	1400	Crespo et al. 2018
**Ordesa 2**	42.5943	0.1529	1160	Crespo et al. 2018
**Picos de Europa 1**	43.1445	-4.9267	1070	Crespo et al. 2018
**Picos de Europa 2**	43.1777	-4.9058	760	Crespo et al. 2018
**Picos de Europa 3**	43.1435	-4.9488	1100	Crespo et al. 2018
**Picos de Europa 4**	43.1723	-4.9086	940	Crespo et al. 2018
**Sierra Nevada 1**	36.9615	-3.4188	1790	Crespo et al. 2018
**Sierra Nevada 2**	37.1838	-3.2628	1710	Crespo et al. 2018
