# Supplementary material for: Optimal inventorying and monitoring of taxonomic, phylogenetic and functional diversity
Source: PLoS One. 2024 Jul 31;19(7):e0307156. doi: 10.1371/journal.pone.0307156 (PMC11290677; doi:10.1371/journal.pone.0307156)
Supplement: Attachment — Submitted filename: PLoS_Reply.docx [file pone.0307156.s003.docx]

Reviewer #3: I believe that it is important to define from the beginning of the methodology what bias is.

Authors: This is now defined in lines 153-154.

R3: On line 228, the bibliographic reference must be placed again for the work where the location and date data of the studies are found.

A: Re-reading this we think this sentence is not needed here as the studies were not presented yet, decided to remove it.

R3: In the discussion, line 430, I suggest reconsidering the statement about the subjective opinion of the researchers, given that I consider that this is a factor that can certainly always have a very positive impact on the development of sampling and studies in general on biodiversity. I do not think it should be rejected and I think it can complement the proposal of the manuscript.

A: Agreed and sentence was modified accordingly.
